# A Systematic Study on Polymer-Modified Alkali-Activated Slag–Part II: From Hydration to Mechanical Properties

**DOI:** 10.3390/ma13153418

**Published:** 2020-08-03

**Authors:** Zichen Lu, Jan-Philip Merkl, Maxim Pulkin, Rafia Firdous, Steffen Wache, Dietmar Stephan

**Affiliations:** 1 Department of Civil Engineering, Technische Universität Berlin, 13355 Berlin, Germany; zichen.lu@tu-berlin.de (Z.L.); rafia.firdous@campus.tu-berlin.de (R.F.); 2 BASF Construction Solutions GmbH, 83308 Trostberg, Germany; janphilip.merkl@gmail.com (J.-P.M.); maxim.pulkin@mbcc.com (M.P.); steffen.wache@mbcc.group.com (S.W.); 3 Bind-X GmbH, Am Klopferspitz 19, 82152 Planegg, Germany

**Keywords:** polymer, alkali-activated slag (AAS), hydration, mechanical properties

## Abstract

The effect of styrene-acrylate (SA) polymer latex on alkali-activated slag (AAS) was systematically studied in the aspects of hydration, hydration products, pore structure and mechanical properties through the combined analytical techniques including calorimetry, X-ray diffraction, thermogravimetric analysis, mercury intrusion porosimetry, and mechanical measurement. It was found that the addition of SA does not retard the AAS hydration, but slightly accelerates it, possibly due to the increasing ion diffusion through the loosely structured hydration products. Pore structure analysis indicates that the addition of polymer increases the cumulative pore volume and the portion of pores with size >100 nm in the hardened AAS paste. The addition of SA latex results in a continuous decrease of the compressive strength, but the flexural strength firstly increases and then decreases with the increase of polymer dosage. The polymer dosage of 2.5 wt % is optimal when applying polymer latex in the AAS system in this study.

## 1. Introduction

Traditional cement-based construction accounts for a large share of environmental CO_2_ emissions and is one of the major causes of global warming. The urgent need for the development of environmentally friendly construction materials and reduction in CO_2_ footprint of cement industry led to the formation of new binders. Alkali-activated material (AAM) represents an alternative cementitious binder due to some specific merits, such as low energy costs, high strength, excellent durability, and low carbon dioxide emissions [1,2,3,4]. A careful design of alkali-activated materials can lead to CO_2_ reduction of up to 30–80% [2]. Henceforth, investigations in the field of AAM have been attracting increasing attention. However, some challenges still need to be addressed to better develop AAM, including efflorescence, high shrinkage, sensitivity to carbonation, periodic variation in the raw materials, lack of standards in an application, etc. [1]. The same problems are also faced during the application of ordinary Portland cement (OPC). However, the broad applicability of chemical admixtures, for example, superplasticizers for modifying the rheology/workability of cement paste [5,6], accelerators or retarders for controlling setting time [7,8], or polymer latexes for enhancing adhesion, anti-shrinkage or anti-cracking behaviour [9], has provided numerous feasible methods to solve these problems. It must be acknowledged that at present well-performing chemical admixtures for overcoming the drawbacks of AAM are urgently needed, and much more effort is required in this field.

Facing this problem, many studies have been conducted on the application of varied chemical admixtures in an AAM system, including superplasticizers [10,11,12], shrinkage reducing agents [11,13], retarders [14,15], however none of these admixtures has made it to the market yet, likely because of low market penetration and higher diversity and complexity of AAM compared to OPC-based systems [16]. Aqueous or powdered polymer dispersions, such as styrene-butadiene rubber (SBR), styrene acrylate (SA) or ethylene-vinyl acetate (EVA) represent a distinguished group of chemical admixtures applied in cementitious materials to improve the flexibility, adhesion, ductility, cracking resistance, impermeability and durability of hardened cement mortar and concrete [17,18,19]. However, only a few papers have been published on the application of polymer latexes in AAM systems [20,21,22,23]. With the addition of styrene-butadiene (SB) latex at various dosages to sodium water glass activated fly ash/slag, Lee et al. [21] found that both flexural and compressive strength of hardened AAM are reduced but the ratio of flexural strength to compressive strength increases significantly, which suggests that the addition of polymer has a higher negative effect on compressive strength than on flexural strength. Kusbiantoro et al. [22] evaluated the effects of poly(ethylene-co-vinyl acetate) (PVA) on the mechanical strength of sodium water glass activated fly ash cured under high temperature (70–90 °C) and found nearly no improvements on the strength of the geopolymer with the incorporation of PVA when the curing temperature is under 80 °C. However, strength can be significantly increased after curing at 90 °C, which is believed to result from the dense microstructure caused by the addition of the polymer. Ribeiro et al. [20] found similar effects on the improvement of strength with the increasing temperature. Besides, the compressive strength of metakaolin activated by potassium silicate and potassium hydroxide firstly increased and then decreased with the increasing dosage of polymer. Hafez et al. [23] concluded that the addition of polymer (styrene-butadiene rubber (SBR) latex and acrylic ester (AE)) results in a significant reduction in drying shrinkage and an improvement in both compressive and flexural strength.

From the literature art above, it is clear that much attention has been paid to the effects of polymers on the mechanical properties of AAM, but no unified conclusion on how the addition of polymer affects the mechanical performance of AAM was given until now. It is commonly accepted that mechanical performance is closely related to the hydration process of cementitious materials and their corresponding hydration products, including the type, amount and morphology. Unfortunately, a systematic study on the effects of polymers on AAM systems from the early reaction to the later mechanical properties is lacking. Moreover, it is found that the activators with different modulus show a vast effect on the polymer colloidal stability [24]. In some cases, the polymer particles lose their colloidal stability immediately after contacting water glass. In order to alleviate the influence of unstable polymer in the AAM system, the different addition methods of polymer latex into the AAM, namely the delayed addition, normal addition, and pre-addition, were applied and their effect on the mechanical properties of hardened alkali-activated slag (AAS) paste was tested by using two types of water glasses (sodium water glass (SWG) and potassium water glass (PWG)). Subsequently, the effects of polymer latex on SWG activated slag were systematically investigated from the aspects of hydration, pore structure and mechanical properties, which aims to build an overall picture of the application of polymer latex in the SWG activated slag system.

## 2. Materials and Methods

### 2.1. Materials

**Figure 1:**
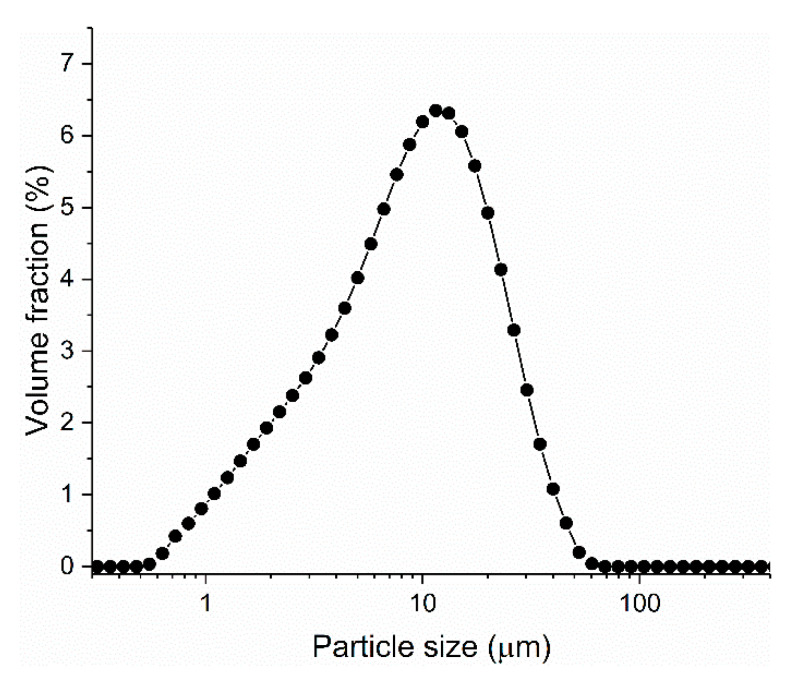
The particle size distribution of slag.

The chemical composition of the ground granulated blast furnace slag used in this study is shown in Table 1. The particle size distribution of slag is shown in Figure 1. The median diameter of the slag was about 14 μm. The initial zeta potential of slag paste, with water to slag ratio of 0.5, was about −12 mV.

One sodium water glass (SWG) and one potassium water glass (PWG) (both provided by Woellner GmbH, Ludwigshafen, Germany) with the silica moduli (M) of 1.81 and 1.02 respectively, were used in this study. Furthermore, one cement-stable styrene-acrylate (SA) polymer latex (obtained from BASF SE, Trostberg, Germany) was used. The SA dispersion was stabilized by PEG-containing surfactants. The underlying physical parameters of SWG, PWG, and the polymer dispersion are shown in Table 2 and Table 3. Deionized (DI) water was used in all the experiments in this study.

### 2.2. Sample Preparation

#### 2.2.1. Polymer Addition Method

The effect of polymer stability on the AAM system was evaluated by using the different polymer addition methods. Depending on the addition sequence of water, water glass, and polymer dispersion, three different polymer addition methods were tested in this study, namely the pre-addition, normal addition, and delayed addition. The pre-addition method means that the polymer dispersion was firstly mixed with water glass and water. After keeping the mixture for 1 d, slag was added into the mixture and thoroughly mixed to prepare the AAS paste. According to our previous results, nearly 50% of polymer particles lost their colloidal stability and coagulated together during the standing period [24]. The normal addition method means that all of these mentioned ingredients were added independently into slag powder and then started to mix immediately to prepare the AAS paste. The drawback of this method was that some polymer particles lost their stability after contacting with water glass and then flocculated, especially when the mass ratio of Na_2_O to slag was above 2.5 wt %, which has been investigated in [24]. Thus, the polymer particles cannot distribute homogenously in the AAS paste and can affect the later performance of the hardened AAS. In order to solve this problem, the delayed addition method was implemented and was compared to the normal addition method. Delayed addition means that water, water glass, and slag were thoroughly mixed and kept for 5 min. Afterwards, the polymer dispersion was added to the paste and mixed again for another 1.5 min.

#### 2.2.2. Sample Preparation

Alkali-activated slag (AAS) pastes with the different added amounts of SA were prepared by keeping the water to slag ratio of 0.5. The mass ratios of M_2_O to slag were chosen to be 2.5 wt % and 3.5 wt % (M corresponds to the alkali metals, namely Na and K in this study), where the mass ratios of SWG to slag were 6.9 wt % and 9.6 wt % respectively (corresponding to a Na_2_O to slag mass ratio of 2.5 wt % and 3.5 wt %) and the mass ratio of PWG to slag was 6.2 wt % (corresponding to a K_2_O to slag mass ratio of 2.5 wt %). Typical dosages of SA (mass ratio of SA to slag) used in cementitious material from 0 wt % to 10 wt % were used in this study. It should be mentioned that the water contained in the polymer dispersion and the water glass should be considered in calculating the water amount which needs to be added additionally. Furthermore, as the void content in the hardened paste has a significant effect on the finally measured mechanical strength, and the addition of polymer potentially introduces more air into the pastes, a certain amount of defoamer should be added along with the addition of the polymer dispersion. In an earlier experiment, we found that the addition of defoamer (PerFin-300, provided by SIKA, Leimen, Germany) at a dosage of 0.08 wt % (by weight of slag) significantly increased the paste density to be comparable to the reference when the dosage of SA was as high as 10 wt %. Hence this dosage of deformer was used in all the prepared samples. The measured density proved a similar void content for each sample. Consequently, the effect of the void content on the mechanical properties of hardened AAS was eliminated. The three addition methods were used to prepare samples with a M_2_O to slag mass ratio of 2.5 wt % to see which method was more suitable to prepare the AAS paste. In the next step, the selected method, i.e., the delayed addition method in this study, was used to prepare all the samples for the further investigation. The dimension of the prepared samples for the mechanical measurements was 8 × 2 × 2 cm^3^. The specific composition of the prepared AAS paste is shown in Table 4.

After preparation, the slag pastes were covered by a thin plastic film and stored at a temperature of 20 °C and RH of 60% for 1 d. The samples were then demoulded and moved into a sealed environment with a temperature of 20 °C and RH of 100% for further experiments, including mechanical measurement, X-ray diffraction (XRD), thermogravimetric analysis (TGA), mercury intrusion porosimetry (MIP), etc. The mechanical strength of the prepared samples was firstly measured at different curing times. Subsequently, the same samples were gently cut into small blocks or milled into powder for further measurements, including XRD, TGA, and MIP, etc. Termination of hydration was conducted by immersing the samples into isopropanol for a particular interval, which is determined by the size of the sample [25], and then dried under vacuum for another 3 d. Previous investigation has demonstrated that the solvent-exchange method (by isopropanol) is the gentlest way to preserve the microstructure in cementitious materials [26]. The samples were then stored in a desiccator free of CO_2_ until the measurement.

### 2.3. Methods

#### 2.3.1. Calorimetry

The exothermic processes of the SWG activated slag paste was continuously monitored for 7 d by calorimeter (MC-CAL/100P, C3 Prozess- und Analysetechnik, München, Germany) at a constant temperature of 20 °C. In order to record heat flow accurately at the beginning, the internal mixing method was applied. The delayed addition method was modified here. After mixing the slag with water glass for 30 s, the polymer was added into the paste and then mixed for another 30 s. The compositions of the samples are shown in Table 4. For the sample without the addition of polymer, the total mixing time was also 1 min but was uninterrupted. The zero points in the calorimetric results correspond to the time when the samples were placed inside the device.

#### 2.3.2. X-ray Diffraction Experiment (XRD)

The samples were firstly prepared, as described in Section 2.2.2. After stopping hydration by isopropanol and vacuum drying, samples were ground and measured by XRD (XRD Empyrean, Malvern Panalytical, Malvern, UK) with CuKα (λ = 1.54 Å) for the 2θ range of 5° to 65° with a step width of 0.013°.

#### 2.3.3. Thermogravimetric Analysis (TGA)

The same sample preparation method, as described in Section 2.3.2, was used for the TGA experiment. Approximately 10 mg of the dried powder was heated at 10 °C/min from room temperature to 1000 °C, in the TGA instrument (TG209 F3 Tarsus, Netzsch, Selb, Germany) under a nitrogen atmosphere.

#### 2.3.4. Mercury Intrusion Porosimetry (MIP)

Mercury intrusion porosimetry (MIP) is a commonly applied method for obtaining information about the pore structure of cementitious materials [27,28,29,30], even though some debates exist regarding the misunderstanding of pore volume or structure because of pore shape [31,32] or polymer film coverage [33,34]. A mercury intrusion porosimeter (Porosimeter 2000, Carlo Erba Instruments, Egelsbach, Germany) was used for testing pore size distribution. Firstly, the samples were prepared, as described in Section 2.2.2. After termination of hydration and vacuum drying, the samples were measured with a maximum pressure p_max_ = 200 MPa. For pore size calculation, an assumption of 130° contact angle and a mercury surface tension of 0.48 N/m was made for all the samples [35] regardless of the addition of polymer or not.

#### 2.3.5. Scanning Electron Microscope (SEM)

The morphologies of AAS pastes with and without the presence of polymer were examined at a curing time of 7 d by SEM (GeminiSEM500 Nano VP, ZEISS, Oberkochen, Germany). Furthermore, the different elemental contents in the hardened paste were also detected by energy-dispersive X-ray spectroscopy (EDS). The same sample preparation method as described in Section 2.2.2 was used.

#### 2.3.6. Mechanical Measurement

The compressive and flexural strengths of the prepared samples, after storing for 3 d, 7 d, and 28 d, were measured by Toni Technik Model 2060 (Zwick Roell, Ulm, Germany). The sample preparation process can be found in Section 2.2.2. For each sample, three independent tests were conducted, and the average value was used in this study.

## 3. Results

### 3.1. Effects of Polymer Addition Methods

**Figure 2:**
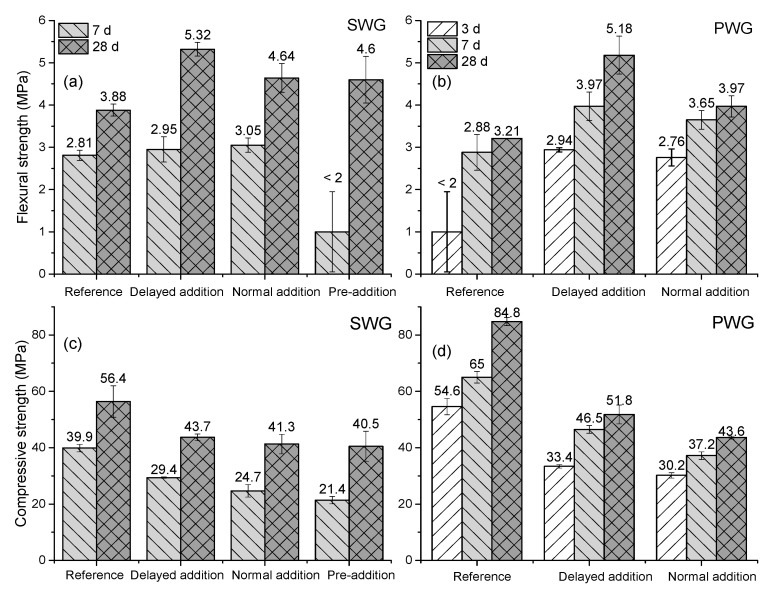
Effects of different polymer addition methods on the mechanical properties of hardened AAS pastes activated by SWG and PWG after curing different times; flexural strength of AAM activated by SWG (**a**) and PWG (**b**); compressive strength of AAM activated by SWG (**c**) and PWG (**d**).

**Figure 3:**
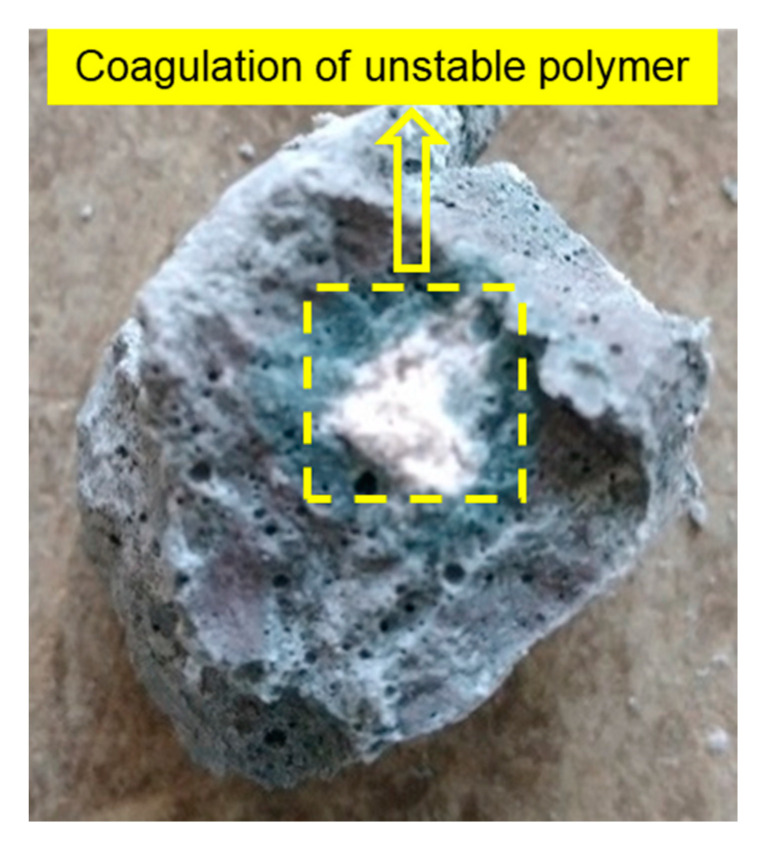
Coagulation of unstable polymer latex in the hardened SWG activated slag paste after curing 7 d.

The effect of three different polymer addition methods on the mechanical properties of hardened AAS paste was evaluated in this part. The AAS paste composition used in this study is shown in Table 4 with the polymer dosage of 0 wt % and 10 wt %, and M_2_O/slag mass ratio of 2.5 wt %. As shown in Figure 2, in comparison to the reference without any polymer, the addition of polymer significantly reduces the compressive strength of the hardened AAS paste, regardless of the addition method. Furthermore, the samples with the pre-addition method show the lowest compressive strength and the delayed addition method always has the highest value, regardless of the different activators used. Similar trends were observed for the flexural strength. The results indicate that the polymer addition method has a significant effect on the mechanical properties of hardened AAS paste, which is closely related to the different colloidal stability and the distribution of polymer particles in the AAS paste. As shown in Figure 3. The unstable polymer particles can coagulate and act as a weak point in the matrix, reducing the strength of the AAM. The delayed addition method is more suitable for the application of polymer dispersions in AAS paste. As a result, only this method was used to prepare samples with different polymer dosages in the following study.

It should be mentioned that the 3 d compressive and flexural strength of slag activated by SWG cannot be measured due to the detection limits of the device. Moreover, the pre-addition method could not be applied for the PWG activated slag because the polymer particles lose colloidal stability immediately after contacting with the PWG. Hence, only the normal addition and delayed addition methods were compared in the case of PWG. Besides, due to device limitations, the exact value of flexural strength <2 MPa cannot be obtained, and it was therefore expressed as a value of 1 MPa, but with an error of 0.95 MPa.

### 3.2. Hydration and Hydration Products

Hydration is the key process to determine the setting and hardening of cementitious materials, and typically the addition of polymer has an enormous effect on the hydration process of OPC [36]. In this study, the effects of polymer on the hydration and hydration products of AAS were evaluated, and the results are shown below.

**Figure 4:**
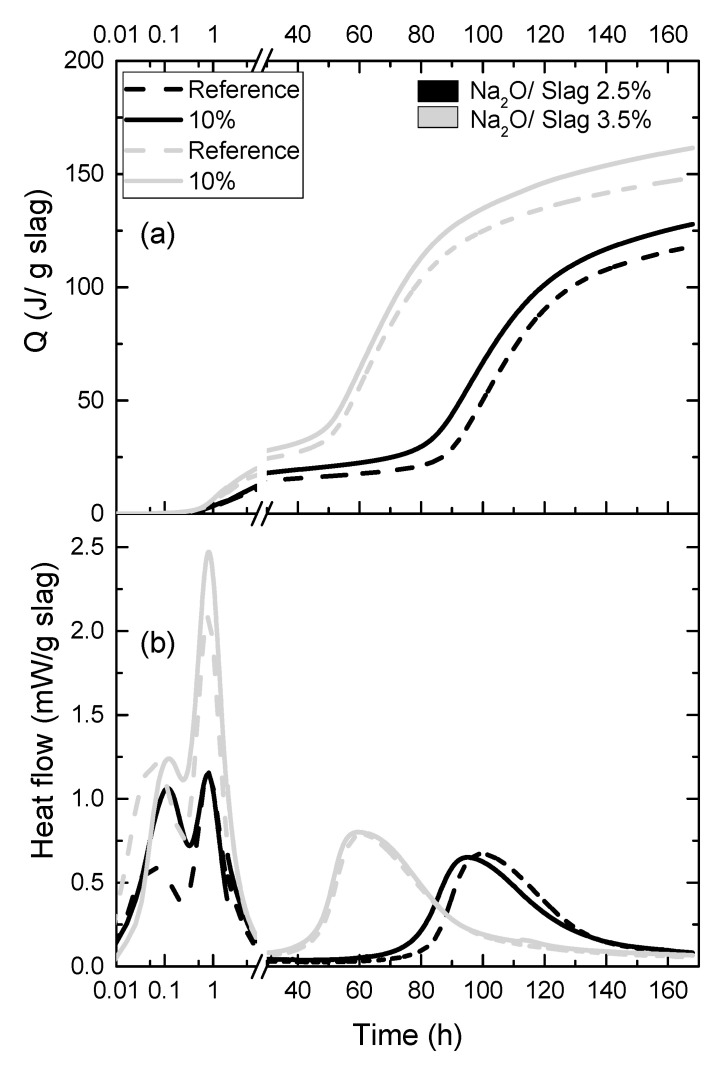
Effect of SA latex at the dosage of 10 wt % on the hydration of SWG activated slag; (**a**) cumulative curves; (**b**) differential curves.

The effects of polymer on the hydration process of the SWG activated slag were evaluated, as shown in Figure 4. Three hydration peaks can be found, one initial peak, one additional initial peak and one main hydration peak. The initial peak is caused by the wetting and dissolution of slag particles [1], and the additional initial peak is originated from the formation of “primary C–S–H” layer at the slag particle surface [37,38]. When the slag grains were coated by a hydrate layer, the hydration process of slag steps into the long induction period, after which the hydration kinetics are controlled by a diffusion process until the completion of the reaction [37,39,40]. Besides, it is reported that the hydration process is mainly affected by the degree of structural defectiveness [37]. Comparing the reference samples with different Na_2_O/slag ratio, the increasing water glass amount significantly increases the height of the initial peak and the additional initial peak, and shortens the induction period, which matches well with the results shown in many literatures [37,41,42]. Regarding the effect of polymer, in contrast to the strong retardation effect of polymer latex on OPC hydration [36], the addition of polymer does not show any retardation effect on the reaction kinetics. On the contrary, it seems that, regardless of the Na_2_O/slag ratio, the addition of polymer leads to slight acceleration on hydration and higher cumulative heat can be found at the same hydration time.

**Figure 5:**
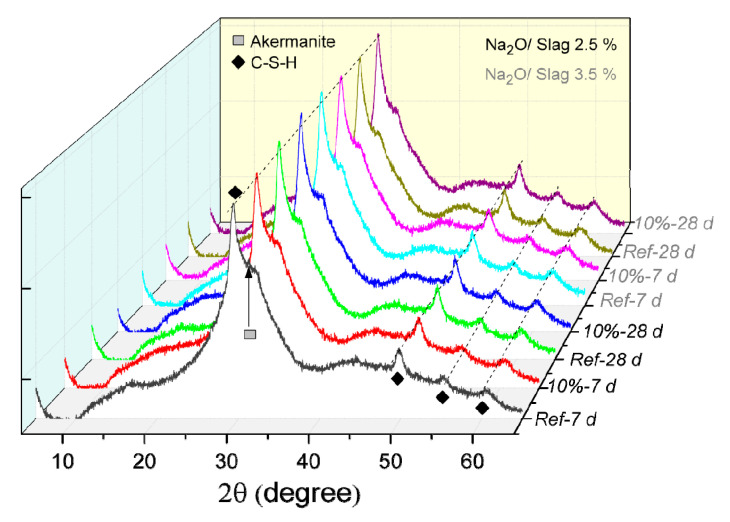
X-ray diffractogram of hardened SWG activated slag pastes with and without polymer (10 wt %) at Na_2_O/slag of 2.5 wt % (in black colour) and 3.5 wt % (in grey colour) after curing certain times (7 d and 28 d).

**Figure 6:**
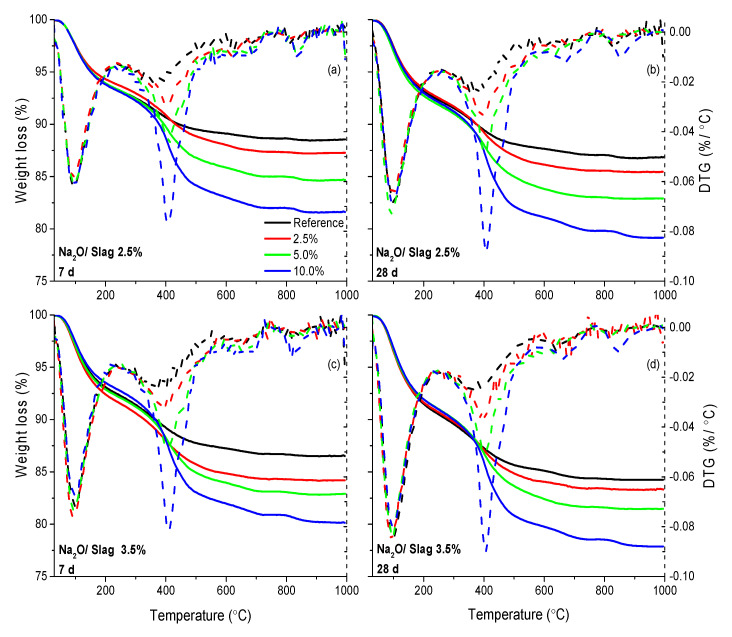
Thermogravimetric results of AAS with the addition of polymer at different dosages and curing times; (**a**) and (**b**): curing time of 7 d and 28 d with Na_2_O/slag of 2.5 wt %; (**c**) and (**d**): curing time of 7 d and 28 d with Na_2_O/slag of 3.5 wt %.

In order to better understand the effect of polymer on the hydration of AAS, the properties of hydration products, including hydration product type, formation amount, morphology, and microstructure are evaluated, as the results shown below. The X-ray diffractogram of hardened AAS paste activated by different Na_2_O/slag ratio with and without the addition of polymer is shown in Figure 5. Firstly, the hydrated samples show distinct diffraction peaks at about 29°, 49°, 55° and 60°, which are attributed to the hydration products of C–S–H. The high peak in the XRD spectra at around 29° indicates a higher crystallinity of C–S–H. The nearly constant C–S–H peak at 7 d and 28 d indicates that the C–S–H formation is quite slow after 7 d. Furthermore, a small peak at about 31° can be found in the diffractograms from hydrated samples shown in Figure 5 and the raw slag (the diffractogram was not shown in this paper), which is assigned to akermanite [43], a mineral phase often occurring in slag. All these phenomena match quite well with the former researchers’ results [44]. Compared to the reference samples, no noticeable difference is found in the diffractograms of the samples with the addition of polymer. It indicates that the addition of polymer does not change the crystalline structure of the formed X-ray detectable hydration products. Due to the effect of a large variety of amorphous phases present in the hardened paste, for example the unreacted slag, XRD results cannot sufficiently characterize the hydration products with or without the addition of polymer. Hence, thermogravimetric analysis (TGA) was applied, and the results are shown in Figure 6. Furthermore, the first derivative of mass losses measured by TGA, i.e., DTG, is also shown in Figure 6.

As shown in Figure 6, for the reference, two distinct peaks were found, corresponding to dehydration of C–S–H (mainly weight loss at 50–200 °C) and hydrotalcite phases (weight loss at around 200 °C and 400 °C) [43]. Besides, two small peaks from the DTG curves were also found at around 650 °C and 850 °C, which are attributed to the decomposition of carbonate phases (formed during solvent exchange process) [45] and the decomposition of C–S–H to wollastonite respectively [46]. It should be mentioned that hydrotalcite was not found in the XRD by the reflection peak but was detected in the TGA analysis curve, which is caused by initial mixing with C–S–H and is consistent with the literature [44]. In our previous experiment, it was found that the polymer decomposes at around 400 °C (as shown in Figure S1). With this information in mind, the comparison was made between the reference sample and samples with the addition of polymer. The weight loss at different temperatures is shown in Table 5.

Regarding the weight loss below 200 °C, it is found that both the increasing Na_2_O/slag ratio and the increasing curing time can slightly increase the values, which indicates more C–S–H is formed. However, if we compare the samples with the increasing polymer dosages, no noticeable difference can be found, which indicates that the addition of polymer does not affect the formation of C–S–H. However, the weight loss at 400 °C increased significantly with the increasing dosage of polymer. It is known that the hydrotalcite phases lose the weight between 200 °C and 400 °C, and the polymer decomposes at around 400 °C (as shown in Figure S1). Hence the decreasing weight between 200 °C and 400 °C should be caused by the overlap of weight loss from hydrotalcite and polymer. In Table 5, in order to contain all the possible weight loss from hydrotalcite and polymer, the weight loss between 200 °C and a higher temperature (500 °C) with and without the consideration of polymer was calculated. It should be noted here that the weight loss from polymer did not simply use the dosage but was measured independently by TGA on the dried mixture of slag and polymer under specific dosages, as shown in Figure S2, and the weight loss caused only by hydrotalcite could be obtained. As shown in Table 5, similarly, the increasing Na_2_O/slag ratio and the increasing curing time can increase the values of weight loss between 200–500 °C, which is caused by the higher hydration degree. However, when comparing the samples with the addition of polymers, the increasing polymer dosage can decrease the weight loss, indicating that less hydrotalcite is formed by the addition of polymer latex.

### 3.3. Microstructure Observation

**Figure 7:**
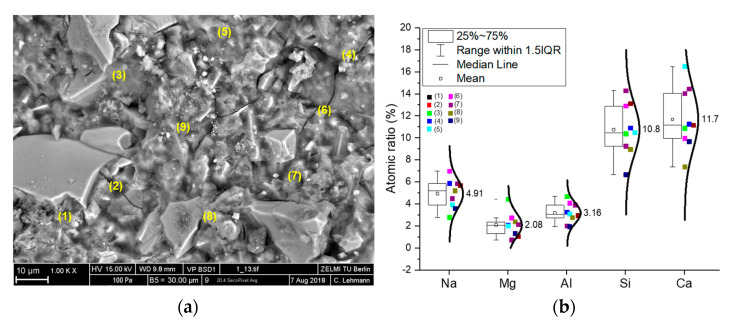
Morphology and elemental analysis of hardened AAS paste without the addition of polymer; (**a**): SEM pictures with different points selected for EDS analysis; (**b**): Atomic ratio of elements in the selected points.

**Figure 8:**
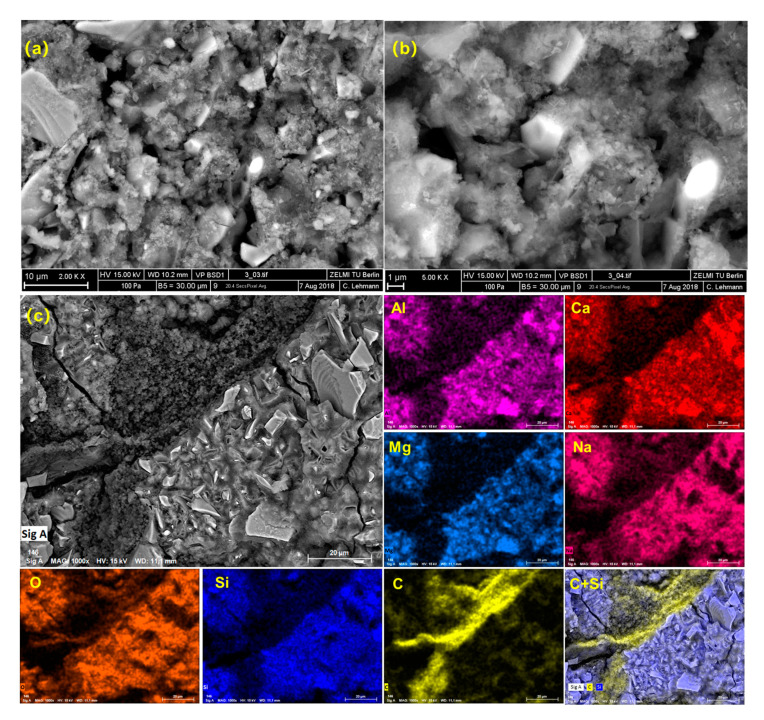
Morphology and elemental analysis of hardened AAS paste with the addition of polymer; (**a**) and (**b**): SEM pictures with different magnifications; (**c**) Polymer films in AAS paste and the corresponding elemental analysis.

Besides the evaluation of the hydration process and the corresponding hydration products with the addition of polymer latex, the microstructure development of hardened AAS paste with and without the addition of polymer latex was characterized through the SEM morphological observation and the EDS analysis, as results shown in Figure 7 and Figure 8. Samples with the polymer dosage of 10 wt % and the ratio of Na_2_O/slag of 2.5 wt % were used for this analysis. As clearly shown in Figure 7a, for the reference sample, a dense C–S–H can be discerned. Based on the EDS results made in specific points, as shown in Figure 7b, the Ca/Si ratio in the formed C–S–H is about 1.1, and a certain amount of Na, Mg, and Al are also incorporated into the formed products, which indicates the formation of C–A–S–H, C(N)–A–S–H [47,48,49]. It should be mentioned here that, in the legend of Figure 7b, IQR means the interquartile range, namely the range between 25th and 75th percentiles (as shown in the area in the box). The error bar shows the range within 1.5 times of IQR. The median line indicates the 50th percentile and the mean value is calculate based on data from all the samples within the 1.5 IQR range and does not include outliers.

To the samples with the addition of polymers, as shown in Figure 8, firstly a loose structure with many tiny pores in the matrix can be observed in the hardened AAS paste. Besides, a region with different morphologies can be found in Figure 8c. Through the elemental analysis supported by EDS, it is found that a large amount of carbon is concentrated in this region compared to the other areas where are full of Si, Al, Ca, Mg, and Na. The concentrated carbon in this area finally leads to a visible polymer film around the slag and/ or their corresponding hydration products.

### 3.4. Mechanical Performance

**Figure 9:**
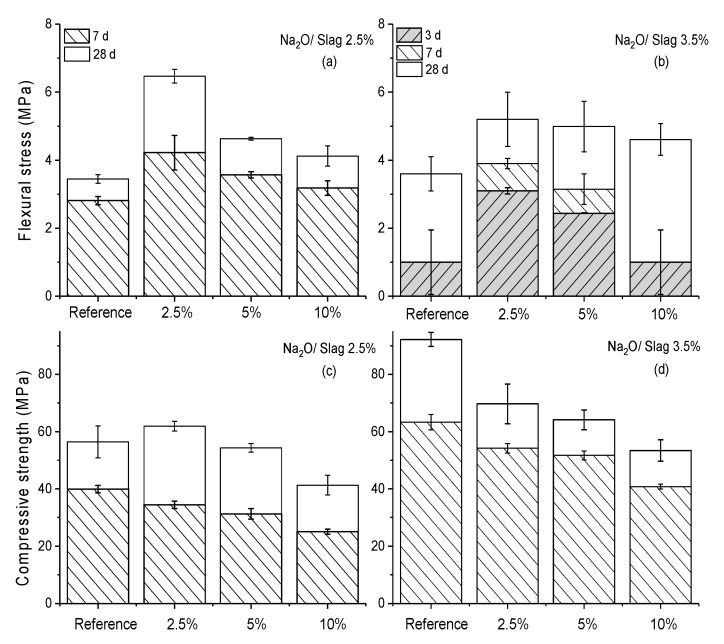
Mechanical properties of AAS paste with the addition of different amounts of polymer after curing certain times; (**a**) and (**b**): flexural strength of AAS with a Na_2_O/slag of 2.5 wt % and 3.5 wt %; (**c**) and (**d**): compressive strength of AAS with a Na_2_O/slag of 2.5 wt % and 3.5 wt %.

The effect of polymer latex on the mechanical performance of hardened AAS paste was investigated, as shown in Figure 9. Increasing dosages of polymer from 0 wt % to 10 wt % were applied in the SWG activated slag under Na_2_O/slag mass ratios of 2.5 wt % and 3.5 wt %.

For the reference activated by SWG with a Na_2_O/slag mass ratio of 2.5 wt %, the compressive strength was approx. 40 MPa and 60 MPa after curing for 7 d and 28 d, respectively. However, the early strength (before 3 d) cannot be measured due to the low value below the detection limit of the device. For the reference activated by SWG with a Na_2_O/slag mass ratio of 3.5 wt %, the increasing SWG dosage significantly promoted compressive strength at every curing time, but it had little effect on flexural strength. The flexural strength after curing for 7 d was decreased compared to the sample with a Na_2_O/slag mass ratio of 2.5 wt %. It should be mentioned here that the exact flexural strength cannot be obtained when it was lower than 2 MPa because of the device limitations. It was therefore expressed at a value of 1 MPa, but with an error of 0.95 MPa, as shown in Figure 9b.

Compared to the reference sample, regardless of the Na_2_O/slag mass ratio of 2.5 wt % or 3.5 wt %, the addition of polymer usually leads to a decrease in compressive strength and an increase in flexural strength, which matches well with the phenomena observed in the case of ordinary Portland cement [50]. However, it is interesting to note that increasing polymer dosages from 2.5 to 10 wt % resulted in a decreased flexural and compressive strength, which has also been reported in the literature [23]. This was quite different to our expectations regarding the effects of polymer on the flexural strength of polymer modified cement paste, in which the flexural strength is usually increased along with the increasing dosage of polymer latex [50].

## 4. Discussion

Based on the results above, two distinct phenomena are found compared to our present knowledge on the effect of polymer latex on OPC. Firstly, instead of the strong retardation of polymer latex on OPC hydration, no retardation, sometimes even a slight acceleration can be found on the AAS hydration process with the addition of polymer latex. Secondly, the increasing dosage of the polymer causes the flexural strength of cured AAS paste firstly increasing and then decreasing, which is different from the continuous increase of flexural strength with the increasing polymer dosages in the hardened OPC paste.

To the effect of polymer latex on OPC hydration, the adsorption of polymer on the cement grains plays a key role in their retardation effect, which can hinder the dissolution of clinkers and the precipitation of hydration products. In the case of AAS hydration, just after the mixing of water glass with slag, the Ca–O, Mg–O, Si–O–Si, Al–O–Al, and Al–O–Si of the slag grain starts to break. Then the primary hydration products start to cover the slag grain. Subsequently, the dissolution of slag and the diffusion of the dissolved ions start to become difficult, which results in the occurrence of the induction period. After that, the hydration process is controlled by the diffusion of dissolved ions, and the degree of structural defectiveness can significantly affect the diffusion process [39,40]. In the presence of polymer latex, the initial formation of hydration products was not hindered by the polymer latex indicated by the height of initial and additional initial hydration peak (Figure 4). Furthermore, because of the loosely micro-structured hydration products (Figure 8) and the less formed hydration products (as shown in Table 5), the diffusion of the dissolved ions can be improved and then a slight acceleration of AAS hydration caused by the addition of polymer can be expected (Figure 4). It should be mentioned here that some researchers concluded that the different adsorption rates of various admixtures on the surface of slag particles could also lead to the acceleration of AAS hydration [51]. Due to the same materials used in this study, this mechanism is not suitable in our case.

**Figure 10:**
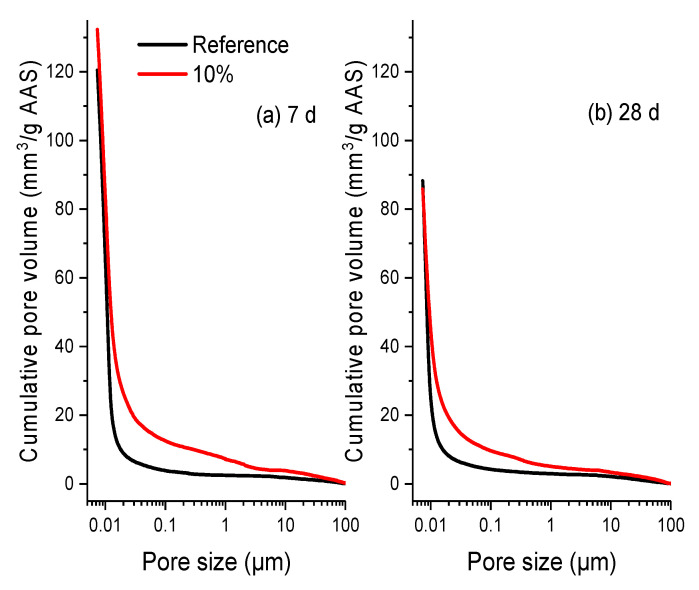
Cumulative pore volume distribution of per unit mass of AAS* with a Na_2_O to slag mass ratio of 3.5 wt %, as detected by MIP (**a**) 7 d; (**b**) 28 d (* The mass of polymer was excluded).

To the effect on the mechanical properties, firstly the pore structures of hardened AAS paste with and without the addition of polymer at the dosage of 10 wt % were characterized through the measurement of MIP. The relationship between the cumulative pore volume (mm^3^/g) and the pore size (μm) per unit mass of AAS (here the mass of AAS means the mass only originated from slag, activator, and water, the mass of polymer was excluded) at different ages (7 d and 28 d), is shown in Figure 10. For the reference sample, the total cumulative pore volume at 7 d is about 120 mm^3^/g. The increasing curing time significantly decreases the volume to about 88 mm^3^/g. Besides, regardless of the curing times, most of the pores in the reference sample have a size in the range of 0 to 100 nm, which corresponds well with the dense microstructure observed in the reference sample, as shown in Figure 7. Compared to the reference sample, the addition of polymer greatly increases the cumulative pore volume to about 132 mm^3^/g after curing 7 d. Besides, the portion of large pores with size >100 nm is greatly increased, which can be proved by the loose structure in Figure 8 with many tiny pores in the matrix. Undoubtedly it is harmful to the mechanical properties of hardened AAS [52]. After curing 28 d, even though the addition of polymer does not have much effect on the total pore volume of the hardened AAS, it again significantly increases the portion of large pores. The increasing pore volume is not beneficial to the mechanical properties of hardened AAS paste.

Besides, as shown in Figure 8, a polymer film is found in the hardened AAS paste. Usually the inclusion of polymer latex in cementitious materials decreases the compressive strength because of the low mechanical property of polymer film compared to that of cement paste [50]. However, the formed film, on the other hand, is believed to be beneficial for the improvement of flexural strength due to the increasing bonding interactions within the matrix [21].

Based on the analysis above, the opposite effect of polymer on the compressive and flexural strength of hardened AAS paste can be explained through the polymer film formation and the change in microstructure. Firstly, the addition of polymer latex can induce the formation of polymer film, which is beneficial for the improvement of flexural strength. However, with the increasing dosage of polymer (dosage of 5 and 10 wt %), the side effect of polymer film on the mechanical properties, the increasing pore volume and the continuous decreasing hydration product amount lead to the reduction on both compressive and flexural strength.

At last, it should be noted that due to the different types, composition and production methods of slag, a huge different performance of AAM can be found by using the same formula but different slags [53,54]. Also, the effect of polymer latex on the properties of AAM can be significantly altered. Hence a further investigation on the dependency of polymer performance on the variety of slag is needed in future study.

## 5. Conclusions

The effects of polymer latex on the hydration process, hydration products, microstructure, and mechanical properties of AAS were systematically studied. The importance of polymer stability on the application of polymer in the AAM system was stressed. Delayed addition is more suitable for the application of polymer latex in the AAS system. The effect of polymer latex on the AAS hydration was first reported. Compared to the retardation effect on OPC hydration, the addition of polymer latex does not retard AAS hydration but slightly accelerate it, which may be caused by the increasing ion diffusion due to the loosely micro-structured hydration products and the decreased formation amount of hydrotalcite. Last but not the least, the incorporation of polymer latex reduces compressive strength but increases flexural strength. The dosage of 2.5 wt % is optimal in this study with the highest flexural and compressive strength. Furthermore, the addition of polymer increases the cumulative pore volume and the portion of large pores in the hardened AAS paste.

## Figures and Tables

**Table 1:** The chemical composition of the slag used determined by XRF (wt %).

Material	SiO_2_	Al_2_O_3_	CaO	MgO	Na_2_O	K_2_O	Fe_2_O_3_	SO_3_
Slag	35.4	13.1	39.8	8.16	0.35	0.62	<0.5	1.17

**Table 2:** Basic physical parameters of the two water glasses.

Samples	Concentration (wt %)	SiO_2_ (wt %)	Modulus	pH
SWG	44.5	28.3	1.81	13.5
PWG	40.0	15.8	1.02	14.3

**Table 3:** Basic physical parameters of the polymer dispersion.

Dispersion	Solid Contents (wt %)	pH	Zeta Potential (mV)	Particle Size d_50_ (nm)	T*_g_*	Stabilizer
SA	53.5	6.05	−22.2	185	−15	PEG-containing Surfactant

**Table 4:** Compositions of AAS paste for different experiments.

Water Glasses	Silica Modulus	Mass Ratio (wt %)					Conducted Experiments
		Water/Slag	M_2_O */Slag	WG/Slag	Defoamer/Slag	SA/Slag	
SWG	1.81	50	2.5	6.9	0.08	0.0	Strength, hydration, XRD, TGA, SEM
						2.5	Strength, TGA
						5.0	Strength, TGA
						10.0	Strength, hydration, XRD, TGA, SEM
			3.5	9.6	0.08	0	Strength, hydration, XRD, TGA, MIP
						2.5	Strength, TGA,
						5.0	Strength, TGA,
						10.0	Strength, hydration, XRD, TGA, MIP
PWG	1.02	50	2.5	6.2	0.08	0	Strength
						10.0	Strength

* M_2_O refers to Na_2_O or K_2_O.

**Table 5:** The weight loss of hardened AAS at different temperature range.

Polymer Dosage	Na_2_O/Slag	Weight Loss (%)					
		7 d			28 d		
		50–200 °C	200–500 °C (With Polymer)	200–500 °C (Without Polymer)	50–200 °C	200–500 °C (With Polymer)	200–500 °C (Without Polymer)
Reference	2.5%	6.2	4.3	4.3	6.9	5.0	5.0
	3.5%	6.9	5.3	5.3	8.6	5.8	5.8
2.5%	2.5%	5.7	6.5	4.0	6.6	6.4	3.9
	3.5%	7.6	6.7	4.2	8.4	6.8	4.3
5.0%	2.5%	6.1	7.1	2.7	7.3	7.7	3.3
	3.5%	7.2	7.9	3.5	8.1	8.3	3.9
10.0%	2.5%	6.2	9.7	1.3	7.1	10.8	2.5
	3.5%	6.5	10.5	2.1	8.1	11.1	2.7

## Supplementary Materials

The following are available online at https://www.mdpi.com/1996-1944/13/15/3418/s1, Figure S1: Thermogravimetric results of polymer, Figure S2: Thermogravimetric results of slag (repeated for 2 times) and the mixture of slag and specific dosages of polymer (repeated for 4 times).
