# Supplementary material for: A Systematic Study on Polymer-Modified Alkali-Activated Slag–Part II: From Hydration to Mechanical Properties
Source: Materials (Basel). 2020 Aug 3;13(15):3418. doi: 10.3390/ma13153418 (PMC7435948; doi:10.3390/ma13153418)
Supplement: Supplementary file 1 [file materials-13-03418-s001.pdf]

materials

Supplementary Materials

A Systematic Study on Polymer-Modified Alkali-Activated Slag–Part II: From Hydration to Mechanical Properties

Zichen Lu, Jan-Philip Merkl, Maxim Pulkin, Rafia Firdous, Steffen Wache and Dietmar Stephan

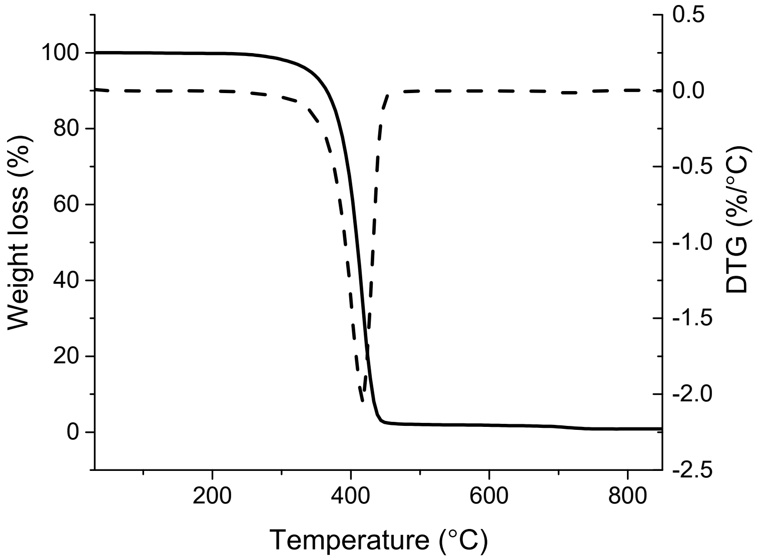

Figure S1. Thermogravimetric results of polymer. The polymer latex was firstly dried into polymer film and then the film was used for the TGA measurement.

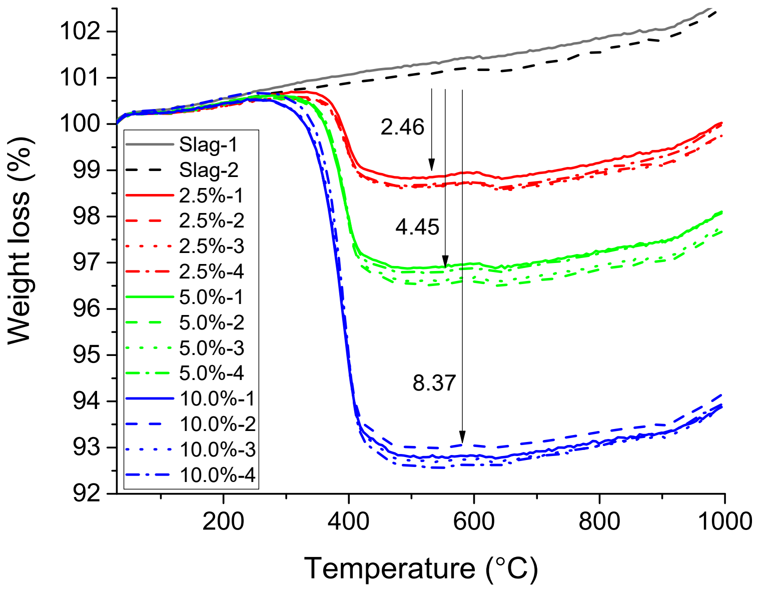

Figure S2. Thermogravimetric results of slag (repeated for 2 times) and the mixture of slag and specific dosages of polymer (repeated for 4 times). The average value of each sample was used for the calculation of polymer contribution to the weight loss before 500 °C. The same sample preparation process as described in Section 2.2.2 was used for these mixtures, including the immersion into isopropanol and the vacuum drying. The measured weight loss caused by polymer is always lower than the theoretical dosage, which may be caused by the removal of surfactant during the sample

preparation process. In addition, the mass of slag always increases along with heating, which may be caused by the sulfur species in slag.

© 2020 by the authors. Submitted for possible open access publication under the terms and conditions of the Creative Commons Attribution (CC BY) license (http://creativecommons.org/licenses/by/4.0/).
